# Association of dietary and lifestyle inflammation scores with muscle strength and muscle endurance among Tehranian adults

**DOI:** 10.1038/s41598-022-23202-z

**Published:** 2022-11-27

**Authors:** Elaheh Asgari, Kurosh Djafarian, Sakineh Shab-Bidar

**Affiliations:** 1 Department of Community Nutrition, School of Nutritional Sciences and Dietetics, Tehran University of Medical Sciences (TUMS), No 44, Hojat-Dost Alley, Naderi St., Keshavarz Blvd, Tehran, Iran; 2 Department of Clinical Nutrition, School of Nutritional Sciences and Dietetics, Tehran University of Medical Sciences (TUMS), Tehran, Iran

**Keywords:** Health care, Risk factors

## Abstract

Diet and lifestyle as modifiable factors play an effective role in muscle strength and muscle endurance. In addition, inflammatory reactions may have an association with the etiology of a a lower muscle strength and muscle endurance. We aimed to investigate the association of dietary and lifestyle inflammation scores (DLIS) with muscle strength and muscle endurance in a sample of Iranian adults. In this cross-sectional study, 270 adults aged 20 to 59 years (55.9% female) were selected. The dietary intakes were collected using a 168-item semi-quantitative food frequency questionnaire. The DLIS was calculated using the dietary inflammatory score (DIS), and lifestyle inflammatory score (LIS). Muscle endurance and muscle strength were measured by a digital-handgrip-dynamometer. Multivariate adjusted means for muscle strength and endurance across quartiles of the DIS, LIS, and DLIS were determined by the ANCOVA test. Multiple linear regression analysis was used to evaluate the association between inflammation scores (i.e., DIS, LIS, and DLIS), and muscle strength, muscle endurance. The DLIS ranged between −2.94 and 3.09. The adjusted P-value of muscle strength of the right hand (MSR) along quartiles of DIS was significantly lower (P = 0.024). MSR (β: −1.19; P-value: 0.020) and mean muscle strength (MMS) (β: −0.95; P-value: 0.047) had significant association with DIS. MSR (β: −0.85; P-value: 0.050) had a marginally significant association with DLIS. Overall, we found that a high adherence to a pro-inflammatory diet might be associated to a lower muscle strength. However, a lifestyle with greater inflammatory potential was not related to any components of muscle endurance. Further studies with prospective designs are needed to confirm the present findings in further details.

## Introduction

Skeletal muscle mass plays an essential role in the strength, function, and regulation of metabolism^1^. Muscle strength and endurance are two separate characteristics of muscle mass^2^. Muscle strength as the ability to produce the most force^3^ is an important tool for assessing nutritional status and a valid indicator of muscle quality and function^4,5^. Muscular endurance as a major part of muscle function^6^ refers to the ability to exert and maintain a continuous force over time^2^. Reduction of muscle strength and endurance can be associated with chronic diseases such as obstructive pulmonary disease, heart disease^7–9^, insulin resistance^10^, and metabolic syndrome^11^. Moreover, assessing muscular endurance along with muscular strength can provide practical information about the ability to perform normal daily activities^12^.

Numerous factors such as genetics^13^, lifestyle habits such as physical activity^14^, alcohol consumption^15^, abdominal obesity^16^, smoking^17^; and dietary patterns^18^, can affect muscle mass. It is proposed that inflammation may mediate the association between the aforementioned factors and muscle mass status^19,20^. To determine the inflammatory potential of the total food intake, the Dietary Inflammation Index (DII) has been designed and validated^21^. However, DII has some limitations and has no indication of the lifestyle^21^.

In a recent study, the use of dietary and lifestyle inflammation scores (DLIS) was validated and recommended^22^. The DLIS contains the dietary inflammation score (DIS) including 19 dietary components, and four components forming the lifestyle inflammation score (LIS): cigarette smoking, physical activity, alcohol drinking, and obesity^22^. Therefore, DLIS can present a relatively comprehensive view of the effects of modifiable factors (diet and lifestyle) on inflammatory status.

Some previous studies have reported that higher dietary and lifestyle inflammatory scores are associated with an increased risk of chronic diseases such as cancer- and cardiovascular disease^23,24^, metabolic syndrome^25^, and insulin-related disorders^26^. However, only a few studies showed that a higher DII score can be associated with reduced muscle mass^27^, muscle strength and endurance^28^, and muscle function^29^. To the best of our knowledge, no prior study has assessed the association between LIS ( with its four components) and muscle strength and endurance. In addition, to the best of our knowledge, no prior work in the Middle East has investigated the relationship between DLIS with muscle strength and muscle endurance. In this study, for the first time, we aimed to investigate the association between DLIS and muscle strength and muscle endurance in Tehranian adults.

## Materials and methods

### Study population selection

In this cross-sectional study, adults were selected from the residents of Tehran (118 males and 152 females). The sample size based on the correlation coefficient can be calculated as the following:^30,31^

1 $$N=\frac{{\left({z}_{1-\frac{\alpha }{2} }+{z}_{1-\beta \times \sqrt{1-{r}^{2}}}\right)}^{2}}{ {r}^{2}}+3,$$

where, the calculated correlation coefficient between pro-inflammatory diets and muscle strength in previous study has been determined as r = 0.37 (the observed correlation)^30,31^. We set the error type 1, α = 5%, and the statistical power, 1-β = 95%, resulting in N = 135. However, due to possible decline in the number of participants, 271 subjects were recruited in this study.

Participants were recruited using advertisements, from February 2018 to July 2019. Inclusion criteria were individuals aged 20 to 59 years old. Exclusion criteria were (i) drug addiction, (ii) having special diets, e.g., weight loss diets and weight gain diets (as they cannot properly represent of the target population), (iii) having acute or chronic diseases which may change the diet routine, (iv) pregnant and lactating women, (v) taking hormone-specific medications, sedatives, supplements, and (vi) any conditions that limit exercise testing (some diseases such as atopy, stroke, asthma, or any other condition that can influence exercise testing). All participants signed informed written consent. The informed consent form and protocol were approved by the ethical standards of the Tehran University of Medical Sciences (ethical number: IR.TUMS.VCR.REC.1397.157).

### Exposures and outcomes

#### Demographic factors

Trained interviewers obtained information about age, gender, education (illiterate, under diploma, diploma, or educated), marriage status (single, married, or divorced), smoking status (never smoker, former smoker, light smoker, or heavy smoker), and occupation (employee, housekeeper, retired, or unemployed). We categorized the smoking status into two groups: “smokers” including current smokers or “non-smokers” including former smokers /never.

### Physical activity

We assessed the physical activity through the validated International Physical Activity Questionnaire (IPAQ)^32^. The obtained data was expressed as a metabolic equivalent minute per week (MET- minute /week)^33^ and accordingly, participants were categorized into three groups: very low (< 600 MET-minute/week), low (600–3000 MET-minute/week), and moderate and high physical activity (> 3000 MET-minute/week)^34^.

### Dietary assessment and calculating the dietary and lifestyle inflammation scores (DLIS)

Using a semi-quantitative food frequency questionnaire, the average food intake in the past year for each participant was obtained^35^. This 168-item questionnaire contained a list of groceries with the standard portion size of each food item. The frequency of consumption for each item was recorded for each subject separately. Utilizing household measures, the portion sizes were converted to grams per day. The questionnaire data was entered into Nutritionist IV software based on the Iranian foods-modified version of the US Department of Agriculture food composition database^36^, and subsequently, we obtained the amount of food intake.

The method introduced by Byrd et al. was used to calculate the DLIS for each participant^22^. The score includes dietary inflammation score (DIS) and lifestyle inflammation score (LIS). The DIS and LIS were calculated as follows. Subsequently, to calculate the DLIS; DIS and LIS for each participant were summed. A higher DLIS indicates a more pro-inflammatory diet and lifestyle.

#### Calculating the dietary inflammation score (DIS)

The DIS includes 19 dietary components including leafy greens and cruciferous vegetables, tomatoes, apples and berries, deep yellow or orange vegetables and fruit, other fruits and real fruit juices, other vegetables, legumes, fish, poultry, red and organ meats, processed meats, added sugars, high-fat dairy, low-fat dairy, coffee and tea, nuts and other fats, refined grains and starchy vegetables and supplement score^22^. Of these, the supplement score was removed due to a lack of relevant information. To calculate the weight of each component in the DIS, each dietary component was treated as a continuous variable (g/d) and then was standardized by gender, to a mean of 0 and SD of 1.0. Next, each dietary component was scored based on the strength of its association with an inflammation biomarker score in the REGARDS case cohort study. Multivariable linear regression was used to estimate the maximum likelihood estimates for the β-coefficients, which represent the average change in the inflammation biomarker score per 1 SD increase in a dietary component. Each dietary component intake was multiplied by the weight (β-coefficient) and then all were summed to calculate the DIS.

#### Calculating the lifestyle inflammation score (LIS)

The LIS includes four components: smoking status, physical activity, alcohol intake, and obesity. We categorized the smoking status into two groups: “current smoker” or “former smoker /never.” Physical activity was divided into two groups of “high or moderate” or “low or no physical activity”. Obesity was characterized by BMI (kg/m^2^) [overweight (25–29.99), or obese (≥ 30)]. Alcohol intake was eliminated due to a lack of information regarding alcohol drinking in the Iranian population. For the LIS, dummy variables were created for physical activity and smoking status, and then multivariable linear regression was applied to estimate the β coefficients to represent the average change in the inflammation biomarker score per having a certain lifestyle behavior relative to its referent category. To calculate the DLIS, the DIS and LIS for each participant were summed. Higher DLIS indicates a more pro-inflammatory diet and lifestyle.

### Anthropometric measurements

The weight, height, waist (WC), and hip circumference (HP) of the study participants were measured by trained dietitians. Subjects were weighted with slight clothing, by an adult’s digital scale (808Seca, Germany) with 0.1 kg sensitivity. The subject’s height was metered using a wall stadiometer with 0.1 cm precision, barefoot with relaxing shoulders (Seca, Germany). Using an anthropometric tape, WC was measured in the middle between the iliac crest and the lower rib margin. The HC was measured by the static tape in the correct horizontal position at the extreme level from the sidelong facet over light clothing, without any force to the body surface with 0.1 cm accuracy. The BMI was calculated as weight in kg divided by height in meters squared. The waist-to-hip ratio (WHR) was calculated by dividing the WC by HP (in centimeters).

### Definition of muscle strength and muscle endurance

Muscle strength and endurance tests were done under the controlled condition: in the laboratory at normal room temperature (23–25 °C), between 8:30–11:30 A.M.^37^. Measures were obtained with a digital handgrip Dynamometer (Saehan, Model SH5003; Saehan Corporation, Masan, South Korea). The validity and reliability of using this instrument have been confirmed^38^. Muscle strength was measured in a standing position while their arms dangling normally on the side of the body and the angle of the elbow was near 180°, subjects were asked to press the handle of the dynamometer as much as possible for measuring the extreme strength for each hand. This method was repeated three times for each hand, entirely six times muscle strength was determined; then in data analysis, a mean of three measurements was applied^39^. For determining muscle endurance participants were asked to continue 1/3rd of maximal deliberate contraction on the condition that he/she could while using a halt watch, the time was recorded^40^.

### Statistical methods

We performed the statistical analyses using the Statistical Package for the Social Sciences (SPSS version 25; SPSS Inc). P-value < 0.05 was considered as a statistical significance level. Participants were evaluated in quartiles of the DLIS. Quantitative variables were reported as mean and standard deviation (SD) and qualitative variables as frequency (percent). The Chi-square test was applied to compare the frequency of qualitative variables across the DLIS quartiles and the Analysis of Variance (ANOVA) was used to compare the means of quantitative variables. Values of daily food consumption across the DLIS quartiles were adjusted for age, sex, energy intake, marriage status, occupation, and education using an analysis of covariance (ANCOVA). Multivariate adjusted means for muscle strength and endurance across quartile of the dietary and lifestyle inflammation scores for the participants were determined through the ANCOVA test, which was adjusted for sex, age, energy intake, marriage status, occupation, and education status. Multiple linear regression analysis was used to evaluate the association between DIS, LIS, DLIS and muscle strength and endurance. We performed this association analysis before and after adjustment for age, sex, energy intake, marriage status, occupation, and education status. For the association of DIS and outcomes, further adjustments for body mass index, physical activity, and smoking status were done.

### Ethical approval and consent to participate

The ethical committee approved the present study of Tehran University of Medical Sciences (ethical number: IR.TUMS.VCR.REC.1397.157). All participants filled out informed consent at the beginning of the study.

### Human and animal rights

This study was conducted according to the guidelines laid down in the Declaration of Helsinki and all procedures involving research study participants were approved by the ethics committee of Tehran University of Medical Sciences. Written informed consent was obtained from all subjects/patients.

## Results

Of the initial 271 participants enrolled in the study, eight participants were removed due to insufficient data for one of the variables, which yielded 263 participants (55.9% female) for the present study. The mean ± SD of age and BMI of participants were 36.8 ± 13.2 years and 25.6 ± 4.6 kg/m^2^, respectively. The inflammatory scores: DIS, LIS, and DLIS were ranged from −3.02 to 1.55 (mean ± SD: −0.50 ± 0.90), −0.41 to 2.07 (mean ± SD: −0.43 ± 0.64), and −2.94 to 3.09 (mean ± SD: −0.08 ± 1.11), respectively. The mean ± SD of mean muscle strength and mean muscle endurance of subjects was 30.9 ± 11.7 kg and 123 ± 67.0 s, respectively. Characteristics of the study participants across quartiles of the DLIS are presented in Table 1. The proportion of married persons increased significantly in the top quartile of the DLIS compared with the lowest quartile, but the percentage of university-graduated persons decreased significantly across the quartiles of the DLIS. Values of age, weight, BMI, WC, WHR, body fat, FM, and FFM increased in the top quartile of the DLIS compared with the lowest quartile.

**Table 1:** Characteristics of participants according to quartile of the dietary and lifestyle inflammation score.

Characteristics	Q1 (n = 65) (most anti-inflammatory) −2.94 to −0.89	Q2 (n = 66) −0.88 to −0.14	Q3 (n = 67) −0.13 to 0.58	Q4 (n = 65) (most pro-inflammatory) 0.6 to 3.09	P-value*
Age (years)	33.7 ± 11.3	34.6 ± 12.8	35.7 ± 13.9	42.6 ± 13.2	< 0.001
Height (cm)	167 ± 9.17	168 ± 11.0	168 ± 10.2	166 ± 9.04	0.477
Weight (kg)	65.1 ± 12.1	71.2 ± 16.7	72.5 ± 15.1	80.8 ± 15.4	< 0.001
BMI (kg/m^2^)	22.9 ± 2.83	24.7 ± 3.86	25.3 ± 4.40	29.1 ± 5.18	< 0.001
WC (cm)	83.2 ± 9.15	87.9 ± 12.1	89.0 ± 12.1	97.5 ± 12.5	< 0.001
WHR	0.87 ± 0.05	0.896 ± 0.06	0.89 ± 0.06	0.93 ± 0.06	< 0.001
Body Fat (%)	27.7 ± 7.86	30 ± 8.41	29.6 ± 9.92	34.7 ± 9.94	< 0.001
FM (kg)	18 ± 5.67	21.3 ± 7.97	21.6 ± 9.12	28.5 ± 11.1	< 0.001
Fat Free Mass (Kg)	47.2 ± 11.5	49.8 ± 13.4	50.8 ± 12.4	52.2 ± 10.5	0.018
Sex (%male)	35.4%	40.9%	47.8%	52.3%	0.220
Marriage status (%married)	40%	48.5%	49.3%	73.8%	0.001
Smoking status (%Current)	6.2%	4.5%	7.5%	13.8%	0.219
Occupation (%)					0.071
Employee	47.7%	57.6%	52.2%	53.8%	
Housekeeper	13.8%	13.6%	14.9%	21.5%	
Retired	7.7%	6.1%	4.5%	15.4%	
Unemployed	30.8%	22.7%	28.4%	9.2%	
Physical activity (%)					0.424
No physical activity	36.9%	28.8%	40.3%	41.5%	
Moderate or heavy	63.1%	71.2%	59.7%	58.5%	
Education (%university graduated)	80.0%	87.9%	67.2%	61.5%	0.002

BMI, body mass index; Q, quartile; WC, Waist Circumference; FFM, fat-free mass; FM, fat mass; WHR, waist-to-hip ratio.

The values are presented as mean ± SD for continuous variables and percent for categorical variables.

*ANOVA test was used for quantitative data and chi-square test for qualitative data. P < 0.05 is significant.

The dietary intakes of participants according to quartiles of the DLIS are indicated in Table 2. By the ANCOVA test, energy intake is adjusted for age and sex, and all other values for age, sex, and energy intake are adjusted. The intake of added sugar, and refined grains increased significantly along with an increase in the DLIS. In contrast, intakes of fiber, tomatoes, apples and berries, deep yellow or orange vegetables and fruit, other fruits and real fruit juices, other vegetables, and nuts decreased proportionally across quartiles of the DLIS.

**Table 2:** Daily food consumption according to quartile of the dietary and lifestyle inflammation score for the participants.

Daily food consumption	Q1 (n = 65) (most anti-inflammatory) −2.94 to −0.89	Q2 (n = 66) −0.88 to −0.14	Q3 (n = 67) −0.13 to 0.58	Q4 (n = 65) (most pro-inflammatory) 0.6 to 3.09	P-value*
Energy (kcal/d)	2289 ± 90.3	2290 ± 89.0	2343 ± 88.2	2367 ± 91.9	0.909
Carbohydrate (g/d)	323 ± 5.96	329 ± 5.88	335 ± 5.82	334 ± 6.07	0.465
Protein (gr/d)	94.1 ± 2.76	87.3 ± 2.72	85.9 ± 2.69	89.5 ± 2.81	0.160
Total fat (g/d)	79.4 ± 2.43	79.4 ± 2.40	76.6 ± 2.38	72.9 ± 2.48	0.204
Fiber (g/d)	17.5 ± 0.51	16.7 ± 0.51	15.8 ± 0.50	13.1 ± 0.52	< 0.001
PUFA (g/d)	16.8.0 ± 0.93	17.1 ± 0.92	16.5 ± 0.91	14.9 ± 0.95	0.403
MUFA (g/d	23.6 ± 0.97	23.6 ± 0.96	22.9 ± 0.95	22.0 ± 0.99	0.619
Leafy greens and cruciferous vegetables (g/d)	28.5 ± 2.56	30.1 ± 2.52	25.8 ± 2.50	20.6 ± 2.61	0.061
Tomatoes (g/d)	28.4 ± 2.21	24.7 ± 2.18	18.3 ± 2.16	15.7 ± 2.25	< 0.001
Apples and berries (g/d)	37.0 ± 2.92	26.1 ± 2.88	24.5 ± 2.86	15.18 ± 2.98	< 0.001
Deep yellow or orange vegetables and fruit (g/d)	50.6 ± 4.17	43.9 ± 4.11	37.7 ± 4.07	17.9 ± 4.25	< 0.001
Other fruits and real fruit juices (g/d)	245 ± 12.8	197 ± 12.6	184 ± 12.5	121 ± 13.0	< 0.001
Other vegetables (g/d)	74.4 ± 4.93	73.3 ± 4.86	65.8 ± 4.82	50.8 ± 5.02	0.004
Legumes (g/d)	68.1 ± 5.43	58.1 ± 5.36	52.5 ± 5.31	55.3 ± 5.54	0.202
Fish (g/d)	0.483 ± 0.21	0.82 ± 0.20	0.381 ± 0.20	0.42 ± 0.21	0.417
Poultry (g/d)	1.16 ± 0.21	0.75 ± 0.21	0.61 ± 0.21	0.61 ± 0.21	0.279
Red and organ meats (g/d)	295 ± 28.7	247 ± 28.3	248 ± 28.0	262 ± 29.2	0.618
Processed meats (g/d)	37.6 ± 4.68	33.8 ± 4.61	29.6 ± 4.57	31.2 ± 4.76	0.645
Added sugars (g/d)	533 ± 52.7	709 ± 52.0	636 ± 51.5	973 ± 53.7	< 0.001
High-fat dairy (g/d)	209 ± 21.6	235 ± 20.8	221 ± 20.6	228 ± 21.5	0.841
Low-fat dairy (g/d)	20.3 ± 2.88	18.0 ± 2.84	17.1 ± 2.82	18.9 ± 2.94	0.877
Coffee and tea (g/d)	7.33 ± 0.91	5.65 ± 0.89	6.59 ± 0.88	3.88 ± 0.92	0.054
Nuts (g/d)	27.4 ± 2.38	20.7 ± 2.35	14.4 ± 2.33	12.9 ± 2.43	< 0.001
Other fats (g/d)	18.7 ± 2.24	21.7 ± 2.21	20.6 ± 2.19	12.7 ± 2.28	0.029
Refined grains and starchy vegetables (g/d)	428 + 22.2	474 + 21.9	529 + 21.7	624 + 22.6	< 0.001

Q, quartile.

Data are expressed as mean ± SE.

* Obtained from ANCOVA test. Energy intake is adjusted for age and sex, all other values are adjusted for age, sex, and energy intake.

Multivariate adjusted means for muscle strength and endurance across quartiles of the DIS, LIS, and DLIS for the participants, separately, are presented in Table 3. By the ANCOVA test, potential confounders including age, sex, energy intake, marriage status, occupation, and education status were adjusted. DIS was adjusted further for body mass index, physical activity, and smoking status. The results showed that the mean of MSR in the highest quartile of the DIS was significantly lower compared to the lowest quartile, (P = 0.024). Across the quartiles of LIS and DLIS, we did not observe any differences in the values of muscle strength and muscle endurance components.

**Table 3:** Multivariate adjusted means for muscle strength and muscle endurance across quartile of the dietary inflammation score (DIS), lifestyle inflammation score (LIS), and the dietary and lifestyle inflammation score (DLIS) for the participants, separately.

Variable	Q1 (n = 65) (most anti-inflammatory)	Q2 (n = 66)	Q3 (n = 67)	Q4 (n = 65) (most pro-inflammatory)	P-value*
Categories of the DIS	−**3.02 to **−**1.07**	−**1.06 to **−**0.49**	−**0.45 to 0.14**	**0.15 to 1.55**	
MMS (kg)	31.3 ± 0.85	32.4 ± 0.87	30.9 ± 0.85	29.3 ± 0.84	0.085
MSR (kg)	32.6 ± 0.91	34.0 ± 0.92	32.3 ± 0.90	30.1 ± 0.89	**0.024**
MSL (kg)	29.9 ± 0.87	30.9 ± 0.89	29.4 ± 0.87	28.6 ± 0.85	0.325
MME (s)	122 ± 8.14	125 ± 8.53	135 ± 8.59	114 ± 8.00	0.362
MER (s)	127 ± 6.52	133 ± 6.83	132 ± 6.87	122 ± 6.40	0.614
MEL (s)	117 ± 12.2	117 ± 12.8	138 ± 12.9	107 ± 12.0	0.367
Categories of the LIS	−**0.41 to **−**0.18**	**0 to 0.32**	**0.48 to 0.89**	**0.98 to 2.07**	
MMS (kg)	30.8 ± 0.80	30.3 ± 1.05	30.5 ± 0.76	32.3 ± 1.08	0.558
MSR (kg)	32.2 ± 0.85	32.0 ± 1.12	31.6 ± 0.81	33.4 ± 1.16	0.675
MSL (kg)	29.4 ± 0.81	28.7 ± 1.06	29.4 ± 0.77	31.2 ± 1.10	0.449
MME (s)	128 ± 7.70	124 ± 10.4	117 ± 7.28	127 ± 10.3	0.714
MER (s)	131 ± 6.09	135 ± 8.30	124 ± 5.75	124 ± 8.18	0.680
MEL (s)	124 ± 11.6	114 ± 15.8	110 ± 10.9	131 ± 15.6	0.639
Categories of the DLIS	−**2.94 to **−**0.89**	−**0.88 to **−**0.14**	−**0.13 to 0.58**	**0.6 to 3.09**	
MMS (kg)	32.0 ± 0.87	31.3 ± 0.88	30.6 ± 0.85	29.7 ± 0.89	0.294
MSR (kg)	33.8 ± 0.92	32.5 ± 0.93	31.8 ± 0.91	30.7 ± 0.95	0.142
MSL (kg)	30.3 ± 0.88	30.1 ± 0.89	29.4 ± 0.87	28.7 ± 0.90	0.590
MME (s)	125 ± 8.29	132 ± 8.67	118 ± 8.17	119 ± 8.70	0.630
MER (s)	127 ± 6.49	141 ± 6.79	127 ± 6.40	116 ± 6.81	0.095
MEL (s)	122 ± 12.5	124 ± 13.1	109 ± 12.3	122 ± 13.1	0.820

MMS, Mean muscle strength; MSL, Muscle strength of left hand, MSR; Muscle strength of right hand, MME, Mean muscle endurance; MEL, Muscle endurance of left hand, MER; Muscle endurance of right hand; Q, quartile; S, second.

Obtained from ANCOVA test. all values are adjusted for age, sex, energy intake, marriage status, occupation, and education status. DIS was adjusted further for body mass index, physical activity, and smoking status. P < 0.05 is significant.

Significant values are in [bold].

Results of multiple linear regression on the association of dietary inflammation score (DIS), lifestyle inflammation score (LIS), and dietary and lifestyle inflammation score (DLIS) with muscle strength and muscle endurance are presented in Table 4. The results showed that DIS had a significant association with MSR (β: −1.19; P-value: 0.020) and mean muscle strength (MMS) (β: −0.95; P-value: 0.047). After further adjustment for body mass index, physical activity, and smoking status, the association of DIS and MSR remained significant (β: −1.19; P-value: 0.023). Also, DLIS had a marginally significant association with MSR (β: −0.85; P-value: 0.050) that was disappeared after adjustment for confounders in model 2 (P = 0.092). We did not observe any significant association between LIS and any components of muscle strength and muscle endurance.

**Table 4:** Association of dietary inflammation score (DIS), lifestyle inflammation score (LIS), and the dietary and lifestyle inflammation score (DLIS) with muscle strength, and muscle endurance.

Variable	MSL (kg)			MSR (kg)			MMS (kg)		
DIS	β	SE	P-value*	β	SE	P-value*	β	SE	P-value*
Crude Model	0.33	0.78	0.670	−0.10	0.83	0.901	0.11	0.79	0.885
Model 1	−0.70	0.48	0.147	−1.19	0.50	**0.020**	−0.95	0.47	**0.047**
Model 2	−0.69	0.48	0.154	−1.19	0.52	**0.023**	−0.94	0.48	0.052
LIS									
Crude Model	1.88	1.10	0.089	1.69	1.17	0.151	1.78	1.12	0.112
Model 1	0.50	0.77	0.517	0.01	0.815	0.988	0.25	0.76	0.737
Model 2	0.68	0.76	0.369	0.31	0.82	0.701	0.50	0.76	0.510
DLIS									
Crude Model	0.86	0.63	0.178	0.51	0.67	0.449	0.68	0.64	0.289
Model 1	−0.367	0.41	0.377	−0.85	0.43	**0.050**	−0.61	0.40	0.135
Model 2	−0.29	0.41	0.474	−0.74	0.44	0.092	−0.52	0.41	0.205

	MEL (s)			MER (s)			MME (s)		
DIS	β	SE	P-value*	β	SE	P-value*	β	SE	P-value*
Crude Model	0.37	6.90	0.957	−1.43	3.72	0.700	−0.52	4.66	0.910
Model 1	−0.14	6.75	0.983	−1.67	3.59	0.642	−0.90	4.49	0.840
Model 2	0.24	6.71	0.971	−1.77	3.60	0.622	−0.76	4.47	0.865
LIS									
Crude Model	18.5	9.93	0.062	5.04	5.32	0.345	11.7	6.69	0.080
Model 1	−1.28	10.95	0.907	−6.29	5.74	0.274	−3.80	7.26	0.601
Model 2	0.27	10.7	0.979	−5.24	5.67	0.357	−2.49	7.12	0.726
DLIS									
Crude Model	5.59	5.68	0.326	0.16	3.03	0.958	2.86	3.83	0.455
Model 1	−1.26	5.82	0.828	−3.46	3.05	0.259	−2.36	3.86	0.542
Model 2	−0.47	5.74	0.935	−3.24	3.03	0.286	−1.85	3.83	0.626

MMS, Mean muscle strength; MSL, Muscle strength of left hand, MSR; Muscle strength of right hand, MME, Mean muscle endurance; MEL, Muscle endurance of left hand, MER; Muscle endurance of right hand.

Data were obtained from the Multiple linear regression.

Model 1 is adjusted for age, sex, energy intake, marriage status, occupation, and education status. DIS was adjusted further for body mass index, physical activity, and smoking status.

Model 2 is adjusted for age, sex, energy intake, socio-economic status score (SES) including: marriage status, occupation, and education status. DIS was adjusted further for body mass index, physical activity, and smoking status.

P < 05.0 is significant. P = 05.0 is marginally significant.

Significant values are in [bold].

## Discussion

This cross-sectional study examined the association between inflammation score based on diet and lifestyle with muscle strength and muscle endurance in Tehranian adults. We observed a significant inverse association between DIS and the MSR and MMS. Also, a higher adherence to DLIS was inversely related to the MSR. To the best of our knowledge, this is the first study that examined the relation between DLIS with muscle strength and muscle endurance in the Middle East.

Our results suggest a significant inverse association between pro-inflammatory diet and the MSR and the MMS. This result may be obtained by decreasing the consumption of fruit and vegetables across the quartiles of DIS and increasing the consumption of added sugar, and refined grains along with the quartiles of DIS. A previous study indicates that dietary patterns with high intakes of fruits and vegetables are related to a lower risk of frailty^41^. A prospective study reported that consumption of added sugars had a direct relation with frailty^42^. In addition,, dietary patterns characterized by high consumption of refined grains had a direct relation with several frailty components^43^. In a previous study, the dietary patterns containing a high amount of red/processed meat and butter were associated with a lower muscle strength. However, in contrast, the dietary patterns rich in fruits, fish/seafood, eggs, nuts, and whole grains containing a lower amount of red/processed meats and potatoes can increase muscle strength^44^.

In this study, we did not notice any significant association between the LIS and any components of muscle strength and muscle endurance. A study on a healthy population showed that among lifestyle components only physical activity in freedom times has a positive association with muscle strength^45^. Although, evidence showed smoking has an inverse association with muscle strength too^17^ because smoking impairs the muscle protein synthesis process, and decreasing muscular fiber also boosts expression of myostatin and muscle atrophy F-box^46^. A longitudinal study showed abdominal obesity, no general obesity has an association with augmenting muscle strength decline in men^16^. Because abdominal obesity more than general obesity can reflect inflammatory activity^47^ which leads to a reduction in muscle strength^48^. While a study reported that general obesity can increase the decline in hand muscle strength^49^. However, we did not observe a significant association between LIS and any components of muscle strength and endurance, which may be obtained by excluding subjects with any comorbidities that can be in connection with their lifestyle.

We showed that DLIS had an inverse association with MSR. In line with our results, a longitudinal study indicated higher DII scores had been associated with a higher incidence of frailty, particularly in men^29^. A cross-sectional study presented that a higher DII scores had a relation to greater odds of sarcopenia^50^. Few studies^51,52^ indicated that the Mediterranean dietary pattern which has components for decreasing systemic inflammation^53^ may reduce the risk of sarcopenia. In contrast with our results, a cross-sectional study on children indicated no significant relationship between DII and handgrip strength^54^. Also, a prospective study indicated no relation between higher DII scores and lower handgrip strength^27^. However, evidence shows that by increasing inflammatory cytokines such as CRP, IL-6, and TNF-a; muscle mass, muscle strength, and physical performance decreased^27^. Inflammatory cytokines declined anabolic factors such as insulin and insulin-like growth factor (IGF-1) can down-regulate muscle protein synthesis^55^. Therefore, a high intake of dietary factors with anti-inflammatory properties may have beneficial effects on muscle strength and muscle endurance.

In this study, we indicated that by increasing DIS, right-hand muscle strength decreased. But did not observe a similar association in the left hand. Because continuous use of the right-hand changes its fiber composition and results in different neuromuscular properties of the right-hand muscle in comparison with the left one^56^.

Strengths of this study are: all measurements were accurate because the collection of the information was done by expert dietitians, using valid and reliable questionnaires and cut-offs; and using a new index that considers important inflammation-related lifestyle components to estimate the joint inflammatory properties of the diet and lifestyle.

However, our study has some limitations that must be considered in interpreting the results. Because of the cross-sectional study design, the temporal sequence and causal relationship could not be determined. In addition, similar to any other cross-sectional study, the present investigation is also susceptible to possible biases such as the recall bias as well as the selection bias. Therefore, further studies with prospective designs in this field are necessary. Moreover, in this study, 18 dietary parameters out of 19 parameters were available for calculating the DIS, and the missing supplementary score. Also, from four lifestyle items to calculate the LIS, one factor (Alcohol-intake) was not considered. The error of measurement and wrong classification of participants cannot be separated from the study. Despite adjusting several distortions in this study, the potential effects of the remaining confounders were uncontrollable.

## Conclusions

Overall, we found that a high adherence to a pro-inflammatory diet might be associated to a lower muscle strength. However, lifestyles with greater inflammatory potentials was not related any components of muscle endurance. Further studies with prospective designs are needed to confirm the present findings in further details.

## Data Availability

The datasets used and/or analyzed during the current study are available from the corresponding author on reasonable request.
